# Fraudulent Licenses

**Published:** 1916-11

**Authors:** C. T. Nolan

**Affiliations:** Secretary State Board of Medical Examiners of Georgia


### FRAUDULENT LICENSES.

On the 15th day of December, 1914, Dr. C. W. Miller, of Atlanta, Ga., ex-secretary of the Eclectic Examining Board, was indicted by the grand jury of Fulton County for forgery in two cases, to-wit: signing the names of the other four members of the Eclectic Examining Board to a license issued to L. L. Lightner of Ideal, Ga.; and to a license issued to W. D. Branch of Baxley, Ga. Dr. Miller made a plea of guilty to a misdemeanor on November 6, 1916 in the court of Judge B. H. Hill, of Fulton County, and was fined $100.00 in each case, including the cost. I have in my possession the records of the Eclectic Examining Board. I have the names now of about thirty doctors who have licenses issued to them and dated May 3, 1913, whose names are not on the Eclectic records. There is no question about these thirty men having fraudulent licenses and the State Board of Medical Examiners is still selling these fraudulent licenses, because Willis N. Goodson of Blaine, Ga., and Dewart B. Tallant of Holcomb, Ga., who graduated at the Georgia School of Eclectic Medicine & Surgery in Atlanta in the spring of 1916, have each a license recorded with the clerk of the superior court at Jasper, Ga., which are issued by the Eclectic Board and dated June 6, 1913.

C. T. NOLAN, M. D.,

Secretary State Board of Medical Examiners of Georgia.

